# Integrative radiation systems biology

**DOI:** 10.1186/1748-717X-9-21

**Published:** 2014-01-11

**Authors:** Kristian Unger

**Affiliations:** 1 Research Unit Radiation Cytogenetics, Helmholtz–Zentrum München, German Research Center for Environmental Health, Ingolstädter-Landstr. 1, 85764 Neuherberg, Germany; 2 Clinical Cooperation Group 'Personalized Radiotherapy in Head and Neck Cancer’, Helmholtz–Zentrum München, Ingolstädter Landstr. 1, 85764 Neuherberg, Germany

**Keywords:** Systems biology, Multi-level integration, Radiation biology, Personalised therapy

## Abstract

Maximisation of the ratio of normal tissue preservation and tumour cell reduction is the main concept of radiotherapy alone or combined with chemo-, immuno- or biologically targeted therapy. The foremost parameter influencing this ratio is radiation sensitivity and its modulation towards a more efficient killing of tumour cells and a better preservation of normal tissue at the same time is the overall aim of modern therapy schemas. Nevertheless, this requires a deep understanding of the molecular mechanisms of radiation sensitivity in order to identify its key players as potential therapeutic targets. Moreover, the success of conventional approaches that tried to statistically associate altered radiation sensitivity with any molecular phenotype such as gene expression proofed to be somewhat limited since the number of clinically used targets is rather sparse. However, currently a paradigm shift is taking place from pure frequentistic association analysis to the rather holistic systems biology approach that seeks to mathematically model the system to be investigated and to allow the prediction of an altered phenotype as the function of one single or a signature of biomarkers. Integrative systems biology also considers the data from different molecular levels such as the genome, transcriptome or proteome in order to partially or fully comprehend the causal chain of molecular mechanisms. An example for the application of this concept currently carried out at the Clinical Cooperation Group “Personalized Radiotherapy in Head and Neck Cancer” of the Helmholtz-Zentrum München and the LMU Munich is described. This review article strives for providing a compact overview on the state of the art of systems biology, its actual challenges, potential applications, chances and limitations in radiation oncology research working towards improved personalised therapy concepts using this relatively new methodology.

## Introduction

### Why we need to improve radiation therapy

Radiation therapy is beside chemo-/immunotherapy and surgery part of the standard treatment of many cancers. Worldwide approx. 13 million new cancer cases and approx. 7.6 million cancer-related deaths arise every year, about half of them in the developed countries [1]. About 60% of cancer cases [2] are treatable with radiotherapy - therefore, any improvement of the success of this treatment option comes with a huge potential impact on the absolute number of additionally cured patients. In the case of head and neck squamous cell carcinoma (HNSCC), a tumour entity that is - mostly in combination with chemo- or immunotherapy - predestinated for radiotherapy, the overall 5-years survival rate is only 45-50% [3] while this rather discouraging number is mostly attributable to the high recurrence rate of this type of cancer which in turn is caused by the resistance of tumour cells to the treatment. Resistance in this context is of multifactorial nature and the specific contributions of radiation alone and the concomitant treatments are challenging to be ruled out. However, it is obvious enough that improvement of radiation therapy would have a significant positive impact on overall therapy success. Moreover, radiation resistance causing local recurrence of the tumours [4] can be of intrinsic nature and thereby a matter of predispositions carried by the patient. The other option is that resistance to therapy is acquired in the course of the therapy as a result of tumour cell evolution during which some cells attain properties of resistance against the pressures built during radiotherapy i.e. reduced radiation sensitivity and which then allow them to overgrow cells missing these properties. Hence, in the context of radiation therapy, individual radiation sensitivity seems to be the key feature of tumours and its understanding needs to be addressed when it comes to efforts of improving the efficiency of radiotherapy.

Whilst a number of markers, although not having made it into clinical practice, driving the radiation sensitivity of normal tissue were identified [5], the knowledge on radiation sensitivity associated markers and mechanisms in tumour cells is sparse. But the overall prerequisite for improving the long-term efficiency of raditoherapy is a deep understanding of the underlying mechanisms of radiation sensitivity in tumour cells in order to get a handle on the control of this phenomenon. The approaches that were taken so far and which were mostly based on plain association testing have not revealed any clinically applied key markers or targets for the modulation of radiation sensitivity in radiation therapy, yet. This might be due to the multifactorial nature of reduced radiation sensitivity which is a mixture of stochastic and deterministic effects and most likely the result of intrinsic and acquired alterations of the cells. Therefore, a rather holistic approach that seeks to address this multifactoriality instead of singling out particular factors for investigation is promising to provide the potential of revealing mechanisms and their key players to be targeted - therefore, systems biology approaches may provide the solution here.

### Targeted treatment options combined with radiation therapy

The phenomenon of radiation resistance is frequently seen in tumours that were treated by radiation therapy or concomitant radiation therapy. Although the mechanisms governing radiation resistance are not fully understood there are already some radiotherapy concomitant treatment options that have made it into clinics that specifically target important signalling pathways or the cells surrounding the tumour that are known to have an impact on the radiation sensitivity. A review by Kaliberov and Buchsbaum [6] summarises the molecular mechanisms of cellular response to radiation and which are involved in the acquired generation of radiation resistance: base excision repair, non-homologous end joining or homologous recombination of double-strand breaks or programmed cell death. Another classification of target mechanisms was formulated by Orth et al. [2] who distinguish radiotherapy concomitant targeted treatment options that modulate radiation sensitivity by aiming at the *DNA damage response*, *topoisomerases*, the *apoptosis network*, *cell division*, *heat shock response*, the *EGFR pathway* and the *tumour micro milieu*. While all of those treatment targets and the modulation of them aims at increasing the radiation sensitivity of tumour cells are promising and already partly successful in improving patients’ outcome a real breakthrough was not achieved, yet.

## Systems biology and its potential role in clinical radiation research

As explained in the previous section existing treatment options accompanying radiotherapy already improve prognosis of the patients, new ways of identifying powerful radiation sensitivity modulators have to be explored and systems biology appear to be promising.

### Definiton and meaning of systems biology

The term systems biology is now widely used and there is not one common definition of it. In reality, there are uncountable ways of understanding, explaining and applying systems biology. Concepts as represented in review articles by Hornberg et al. [7] or Bruggeman and Westerhoff [8] nicely describe what could be considered as common understanding of systems biology. In order to make clear how I understand systems biology I suggest one definition of systems biology as follows:

While traditional, reductionistic approaches investigated the property of one or of a few components (i.e. molecules) or their interaction with one or a few other molecules at the time, systems biology investigates the emergent properties of the system under investigation (e.g. organelles, cells, organs, organisms or eco systems) when multiple entities interact in networks. To do so, systems biology requires highly interdisciplinary approaches involving expertise from physics, mathematics, graph network theory and biology that uses all molecular and other data (such as phenotypic data or clinical data) available for integration and the creation of a systems model that is capable of predicting the response of the system to a particular perturbation.

Systems biology for certain has its origin in physics, a discipline that traditionally builds models of what is observed in order to predict the “response” to a perturbation. So it is for example possible to deduct from any circuit diagram of a system consisting of a battery, resistors and LEDs what will happen when any of the included elements are changed in any way (perturbed). The accuracy of the prediction in the case of circuit diagrams is very good, almost perfect for smaller systems, but also very good for highly complex systems. This very high level of accuracy is because we know every single part making up the system and we know how and in which way these elements are connected to each other. Of course, also here we have to accept some inaccuracy, but compared to biological systems technical systems are a almost perfect world. In contrast to technical systems, the behaviour of biological systems, such as cells, organs, tumours, organisms etc. are not very much predictable. This might be due to two reasons: firstly, compared to technical systems we do in the moment not know enough about the elements biological systems are composed of and most importantly we do not know how these work together. Secondly, technical systems are usually composed in a modular fashion which makes even very complex systems controllable and predictable. Whether biological systems are modular as well or not cannot be answered in the moment. So is the “discovery” of epigenetics and post-transcriptional regulation by miRNAs not older than 20 years and we still miss THE explanation for how biological systems and the elements they are composed of really work together. However, there seems to be a significant controversial between the thinking of biologists who have the genuine interest in using systems biology approaches and those who actually have the expertise of designing and applying these approaches. The article “Can a biologist fix a radio?” by Lazebnik [9] nicely describes this situation in an highly informative and truly entertaining way.

Systems biology can be used to find the causal relationships between the elements making up a biological system which are genes, mRNAs, miRNAs, proteins and metabolites - between receptors, transcription factors and phenotypic effects. With regard to the identification of all of these elements we are certainly not far off completion, however we need to understand *how* these work together. And we are trying to do this by applying the methodologies from systems physics to biology. The main steps in getting to a systems model is to identify the network that is affected by the perturbation, to reduce this network to the highest informative elements and to model the response of the network to the perturbation.

Finally, systems biology allows to think in processes rather than in momentary snapshots reflected by single measurements done at random time points. Provided the required time-resolved data are available molecular mechanisms can be described as a function of time. Bechtel [10] generalises this concept and assumes an organism to be composed of oscillating processes and that disruption of these processes in fact leads to diseases. The other way around this rather philosophic point of view in consequence means that we have to identify the processes and the elements they are composed of in order to come to a solution that allows to 'resynchronise’ the disrupted oscillating processes.

### Multi-level data integration

**Figure 1:**
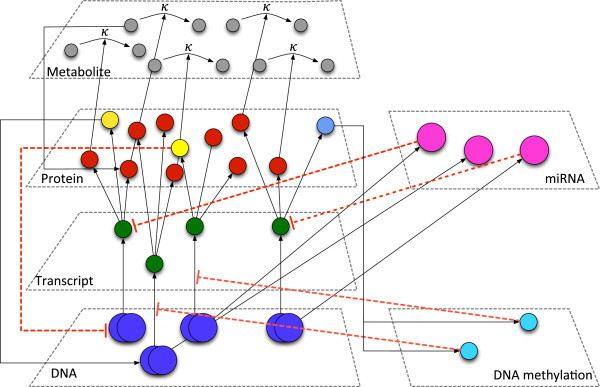
**Integration of multiple omics-level.** Simplified overview of the integration of omics data at the DNA, transcript and protein level and the regulatory miRNA and DNA methylation levels. According to the “central dogma of molecular biology” [34] information is transferred from DNA (genes, blue) to the RNA level (transcripts, green) and to the protein level (proteins, red) in a linear manner. Proteins i.e. enzymes then catalyse biochemical reactions in which metabolites are processed. The metabolites are indicated by grey circles whilst the *κ* sign symbolises that this process follows certain kinetics. The concentration of metabolites is well measured by receptor proteins - therefore, there is a strong communication between the protein and metabolite level. However, both transcription and translation and the lifetime of transcripts and proteins is regulated by other levels such as DNA methylation (cyan) [35,36] and miRNAs (pink) [37,38]. Mediated by transcription factors (yellow) [39,40] there is also a powerful feedback from the protein level back to the DNA level. Another powerful molecular switch are proteins (DNA methyltransferases and DNA demethylases, light-blue) that control transcription by changing the level of methylation of histones - therefore, there is a feedback from the protein level to the DNA methylation level.

**Figure 2:**
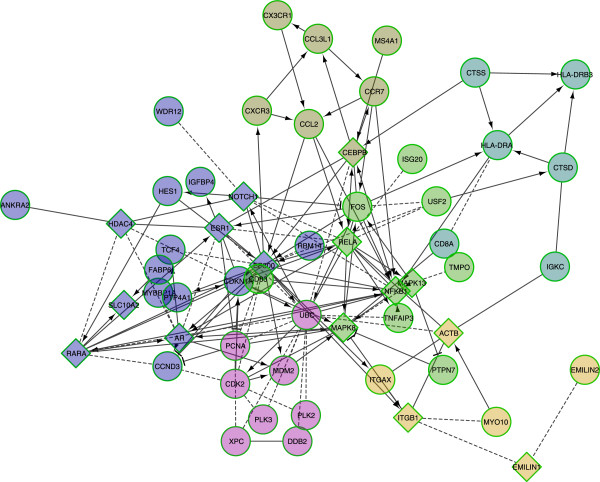
**Reactome FI functional interaction network generated using the differentially expressed genes from a study by Henriquez et al. [**[33]**] comparing global microarray expression profiles of lymphocytes from patients before and after 2Gy X-ray irradiation.** From the 66 HGNC annotated genes (Supplementary table two [33]) 44 were found to be part of an interaction network. So-called linkers (n=15) i.e. proteins not part of the gene list that allow indirect interaction between two genes are indicated by diamond-shape nodes and the genes from the list by circles. Predicted interactions are indicated by dashed black lines and interactions for which experimental evidence exists by solid black lines. Where known the type of interaction is indicated by arrow-headed lines (activating) or by bar-headed lines (inactivating/inhibiting).

Integration of the data from the multiple molecular levels is a subdiscipline of systems biology. The main task of data integration is to identify causal relationships between the different molecular levels (Figure 1). From the resulting data we can learn how the different levels work together and what the causal relationship between a particular perturbation and the phenotype (e.g. radiation treatment of cells and its impact on the survival rate of the cells) is. The most common molecular levels being characterised in molecular studies are the genome level by array CGH or SNP microarray analysis [11,12], the transcriptome and miRNA level by expression microarrays [13,14], global methylation patterns by either hybridisation of methylated sequences enriched by chromatin immunoprecipitation (ChiP, [15]) onto microarrays (ChiP-on-chip [16]) or microarray-based characterisation of the genome-wide methylation status after bisulfite-conversion of genomic DNA [17]. Moreover proteomics methodology allows to characterise the proteome, metabolome or lipidome [18]. One way of identifying causal relationships between different molecular levels is to bioinformatically match the measurements from the levels to be integrated. Matching of data at the genomic level can be achieved by using the genomic location of the probes of each platform - this applies for the integration of DNA methylation profiles with array CGH profiles. If one wants to find possible associations between miRNA expression and the genomic copy number, the location of the probes of the miRNAs microarray need to be matched with that from the genomic positions of the probes of the CGH array ([19,20]). In the case of integration of gene expression with protein expression data the mRNA microarray probes and protein expressions are matched using the gene/protein names. Another extremely important switches in the regulation of gene and protein expression are transcription factors that can be considered as a feedback connection from the protein level to the genomic level (Figure 1). In order to test possible associations between transcription factors and protein/gene expression, genes that are under control of a particular transcription factor need to be either experimentally identified by Chip-seq technology [21] or by *in silico* analysis using algorithms predicting transcription-factor gene relationships based on promoter-affinity analysis [22]. Other ways of integrating multi-level data that not directly match the elements of the different molecular layers is based on graphical models or statistical associations. Two major approaches representing these concepts are gaussian graphical modelling [23] and genotype-environment interaction analysis [24]. Gaussian graphical models are based on partial correlation which assumes that the correlation of two variables is influenced by a third variable. By using this approach it is for example possible to identify gene regulatory networks that are specifically influenced by histone acetylation [25] or to integrate metabolomics with genomics [26]. In the case of genotype-environment interaction analysis the information from genomics, transcriptomics and on the phenotype (e.g. disease) are integrated in such a way that potential targets from the expression level for modulating the phenotype can be identified. Moreover, an important element of multi-level integrative analysis is the use of *a priori* knowledge on interactions using appropriate databases. There is a number of databases and tools around allowing not only pathway enrichment but also topology-based pathway analysis. The development and state of the art of pathway analysis over the last decade from simple gene set overrepresentation analysis over functional class scoring (FCS) approaches to the lastest generation of pathway topology based approaches is nicely reviewed by Khatri et al. [27]. Examples for databases providing protein-protein interactions are http://www.genome.jp/kegg/KEGG[28] or http://www.reactome.orgReactome[29] or http://string-db.orgSTRING[30]. These databases provide interaction networks at a global level or allow to specifically search for interactions of a candidate of interest. If available the interactions or interaction predictions are classified according to their level or source of evidence (e.g. text-mining vs. evidence by two-hybrid assays [31]). A very straightforward tool to match genes of interest to existing knowledge of interaction is the http://wiki.reactome.org/index.php/Reactome_FI_Cytoscape_PluginReactome FI Cytoscape Plugin, a plugin for http://www.cytoscape.orgCytoscape[32] that allows to explore known or predicted interactions between genes or proteins e.g. from a comparative gene expression study and to nicely visualise the results in the form of an interaction network. An example for an Reactome FI analysis with the genes revealed to be differentially expressed in lymphocytes of HNSCC patients before and after therapeutic irradiation [33] is shown in Figure 2.

### Network reconstruction

A lot of effort was put into the development and application of network reconstruction approaches using high-dimensional data for the delineation of molecular interactions. The dimensionality of the dataset is determined by the number of measurements (e.g. the number of probes on a microarray) which is usually much greater than the number of samples, the latter of which determining the maximum rank in a covariance matrix - the basis for many network reconstruction approaches. The smaller the number of samples and consequently the maximum rank, the lower the chance of finding true molecular interactions. In order to address this problem Thomaz et al. suggested the MECS method (maximum entropy covariance selection) for the estimation of covariance [41]. One very remarkable effort to systematically assess the performance of different network reconstruction approaches in different data situations (static measurements from clinical samples vs. dynamic data from time-course perturbation experiments) was the “Network Inference Challenge” of the DREAM (Dialogue for Reverse Engineering Assessments and Methods) initiative. A summary of the approaches and the outcome of this challenge was published in 2012 [42]. The reference for assessing the performance of the methods were the *a priori* known interaction network of S. *cerevisiae* along with mRNA and protein expression data and a dataset consisting of a simulated interaction network along with mRNA array expression data. There are various methods for the reconstruction of gene regulatory networks available and all of them have strengths and weaknesses and to-date there is no one-for-all solution available that works for every data situation. Every method and network that is reconstructed comes with a certain level of uncertainty i.e. false positive and false negative findings. A review article by Karlebach and Shamir [43] gives an excellent and comprehensive overview on the various approaches available and discusses the advantages and disadvantages of each method.

### Graph network theory and network visualisation

The reconstructed gene regulatory networks can be described, visualised and analysed by the so-called graph network theory, a method that is widely used, most importantly in social sciences while social networks are probably the most popular application of network graph theory. Popular network visualisation tools are yED and Cytoscape [32]. The overall principle of graph network theory is to understand the elements (genes, proteins, metabolites etc.) making up a network as nodes or vertices and the connections between them as edges and to mathematically analyse the structure and topology of these networks. Mitrea et al. wrote an excellent and easy to understand review article on this [44]. An important feature of graph network theory is the possibility to analyse the “importance” of any given node or set of nodes by considering the topology of the network they are embedded in. Amongst the most commonly used metrics for this are the so-called centrality measures such as degree, betweenness, closeness, and eigenvector centrality [45].

### Mathematical modelling of “systems” networks

The overall objective of systems biology approaches is to come to a mathematical model that allows to predict the response of the cells to specific perturbations, for example the treatment with a particular drug. The principle of mathematical modelling is to use experimental measurements on the expression of genes or (phospho-)proteins of the nodes composing the network of investigation in order to explain causal relationships between the perturbation and response. In frame of this review it is not possible to go into detail of the commonly used approaches of mathematical systems modelling. However, for this purpose I recommend a review article by Klipp and Liebermeister (2006) that gives a comprehensive overview on the topic [46]. A very basic and central element in modelling gene regulatory networks are so-called ODEs (ordinary differential equations) [47] that basically describe the change of concentrations over time. The rates at which the concentrations change and the mode of kinetics need to be individually chosen for the system to be modelled. The prototype of an ODE being used in the reconstruction of gene regulatory networks is the Hill differential equation [48] that initially was built to describe kinetics of the binding and dissociation of oxygen to haemoglobin. Generalisation of the Hill kinetics is frequently used in the reconstruction of gene regulatory networks. Solving the equation allows to come up with a first prediction which in turn has to be compared with real experimental measurements followed by an iterative adjustment of the initial model in order to come to a final model that allows most accurate prediction of the behaviour of the network in response to any perturbation.

## Promising outcomes from two systems biology studies

Two recent studies on colon cancer [49,50] that systematically modelled the response of the RAS/PI3K signalling pathway to inhibition of EGFR by cetuximab revealed strong negative feedback loops between ERK and EGFR. This feedback loop immediately compensates any EGFR inhibition by cetuximab by activating EGFR. Therefore, only double-inhibition of EGFR and MEK [49] or BRAF [50] seems to break this feedback connection in order to bypass any acquired resistance to cetuximab. Actually, a study on colon cancer is currently trialling a combinatorial treatment of BRAF/MEK and EGFR with Dabrafenib, Trametinib and Panitumumab (www.clinicaltrials.gov, ID:NCT01750918). Referring to the Klinger et al. study [49] a systems model should not be of too high complexity and should be therefore very much reduced to informative nodes only.

These two studies could serve as a role model for tackling the phenomenon of acquired resistance in HNSCC to radiation treatment. The activities of the below described clinical cooperation group are actually also aiming to get to a systems model explaining acquired resistance to radiotherapy in HNSCC and thereby providing molecular targets to break resistance to therapy.

## An example of an application of systems biology in radiation oncology research: the clinical cooperation group “Personalized radiotherapy in head and neck cancer”

**Figure 3:**
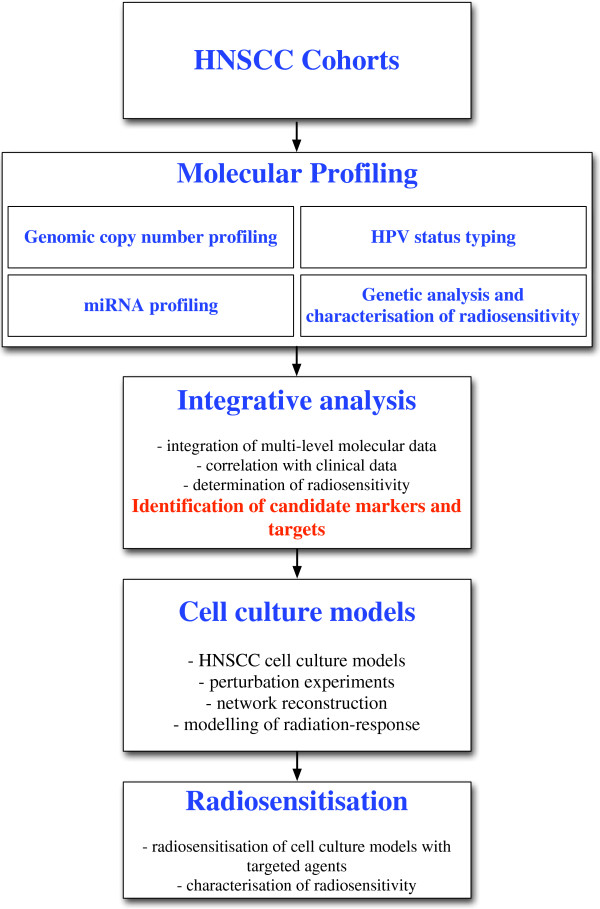
Strategy of the clinical cooperation group “Personalized Radiotherapy in Head and Neck Cancer” implementing systems biology approaches.

The clinical cooperation group (CCG) “Personalized Radiotherapy in Head and Neck Cancer” is a structure aiming at the intimate collaboration of research and oncology in order to understand the mechanisms of radiation resistance in HNSCC and to identify targets that allow to overcome resistance to radiation therapy. The CCG allows intensive exchange of expertise and knowledge between academic and oncology research whilst the clinicians formulate the questions to be addressed by the research carried out and thereby allow the researchers to most efficiently work towards the common goal of improving radiotherapy of HNSCC. The CCG has a core project (Figure 3) which starts with the molecular characterisation of clinical samples from selected HNSCC cohorts. With regard to the global characterisation of molecular levels we focus on the genome and miRNA level in the first place since these are the best accessible ones wenn it comes to analysis of archived clinical samples. The global molecular characterisation data are complemented with typing of the HPV status and individual mutations known to have an impact in HNSCC, with clinical follow-up including endpoints reflecting response of the treated tumours to radiation therapy. These are integrated in order to come to candidate molecules that are likely to be involved in the molecular mechanisms of radiation sensitivity and that can serve as a starting point for time-resolved molecular characterisation at the transcriptome and miRNA level of cell culture models after perturbation by regulating the candidate molecules that were identified in the clinical samples. The resulting global time-course mRNA and miRNA data are then used for reconstructing the interaction networks of the candidate molecules and also allow to infer the molecular mechanisms associated with radiation sensitivity. Different methods are explored and those with the best performance, which will be validated by further experiments (i.e. knock-down of specific genes), will be chosen. Since we are looking at gene regulatory networks that are specifically influenced by genomic copy number and miRNA expression the use of Gaussian graphical modelling [23] might provide the solution here. Further, using graph network analysis the most important (central) nodes that could be used as molecular modulators of radiation sensitivity are identified. The selection of candidates will be performed using well established centrality measures (e.g. betweenness) in order to get to the “most important” molecules of the network. In a second step this selection will be explored for drug “targetability” using established drug databases such as DrugBank [51]. These candidates are then characterised for their radiation sensitivity modulating effect in cell culture models. By following this approach, with integrative systems biology as a central element, we will identify potentially important molecular targets being used for a more efficient radiation therapy resulting in lower rate of tumour recurrence and overall better long-time survival of the patients.

## Limitations of systems biology in radiation oncology research

In radiation oncology research one has to deal with the effects of radiation on cells whilst the main aim is to protect and favour the tissue surrounding a tumour and to harm and delimit the tumour itself as much as possible. Radiation effects are composed of deterministic effects such as cell death or normal tissue reactions and stochastic effects such as point mutations or structural changes or changes in copy number of genes. Whilst for deterministic effects it is possible to predict what effects at which amounts are expected when applying a certain dose of defined radiation quality it is, by definition, impossible to predict the stochastic effects. Although there are systems biology approaches dealing with stochastic effects they cannot predict the occurrence of the primary radiation damage which in the radiation therapy setting are damages at the DNA level. So it remains unpredictable which genes are going be to altered as a cause of radiation treatment in the cell populations of both, the tumour tissue and its surrounding normal tissue, surviving each fraction in fractionated radiation therapy. Logically, the molecular mechanisms associated with the radiation-induced gene alterations cannot be predicted either. Thus, one could doubt the use of systems approaches in radiation oncology research at all. However, major obstacles of successful radiotherapy such local tumour recurrence or radiation resistance seem to be ruled by a set of common molecular mechanisms. For that reason it should be legitimate to assume that the primary effects of radiation indeed are of stochastic nature but that the inter-individual selective pressures effectuated by the radiation therapy and any concomitant treatment are the same between individuals and therefore lead to manifestation of the same set of molecular survival strategies. At this stage the system becomes describable and predictable again. So the prerequisites of successful systems approaches in radiation oncology research are to mimic the *in vivo* situation as best as possible and to “reverse translate” the findings gained in cell culture models to clinical samples HNSCC patients.

## Conclusions

In the context of radiation therapy and the improvement of this therapy option alone or in combination with immuno- or chemotherapy seems to be extremely important taking into account its wide usage and the relatively low success rate with regard to long-term survival. The key feature to be addressed for improving radiation therapy is radiation sensitivity and its modulation towards higher sensitivity of tumour cells and lower sensitivity of the surrounding normal cells. Conventional approaches seeking for potential modulators did not yet provide the breakthrough, therefore implementing systems biology methodology into radiation oncology research is a very much promising approach. The clinical cooperation group “CCG Personalized Radiotherapy in Head and Neck Cancer” between the Research Unit of Radiation Cytogenetics at the Helmholtz-Zentrum München and the Radiation Oncology Clinics of the Ludwig-Maximilians Universität München currently applies a systems biology approach in order to identify candidate radiation sensitivity modulators for improved radiation therapy of HNSCC.

## Competing interests

The author declares that he has no competing interests.

## Authors’ information

Clinical Cooperation Group “Personalized Radiotherapy in Head and Neck Cancer”, Helmholtz-Zentrum München/Ludwig-Maximilians Universität München.
